# Active Fish Gelatin/Chitosan Blend Film Incorporated with Guava Leaf Powder Carbon Dots: Properties, Release and Antioxidant Activity

**DOI:** 10.3390/gels10040281

**Published:** 2024-04-21

**Authors:** Gokulprasanth Murugan, Krisana Nilsuwan, Thummanoon Prodpran, Arunachalasivamani Ponnusamy, Jong-Whan Rhim, Jun Tae Kim, Soottawat Benjakul

**Affiliations:** 1 International Center of Excellence in Seafood Science and Innovation (ICE-SSI), Faculty of Agro-Industry, Prince of Songkla University, Hat Yai, Songkhla 90110, Thailand; gokulprasanth031999@gmail.com (G.M.); krisana.n@psu.ac.th (K.N.); thummanoon.p@psu.ac.th (T.P.); 6511030004@email.psu.ac.th (A.P.); 2 Center of Excellence in Bio-Based Materials and Packaging Innovation, Faculty of Agro-Industry, Prince of Songkla University, Hat Yai, Songkhla 90110, Thailand; 3 BioNanocomposite Research Center, Department of Food and Nutrition, Kyung Hee University, 26 Kyungheedae-ro, Dongdaemun-gu, Seoul 02447, Republic of Korea; jwrhim@khu.ac.kr (J.-W.R.); jtkim92@khu.ac.kr (J.T.K.)

**Keywords:** gelatin, chitosan, carbon dots, UV barrier, antioxidant, water vapor permeability

## Abstract

Active packaging is an innovative approach to prolonge the shelf-life of food products while ensuring their quality and safety. Carbon dots (CDs) from biomass as active fillers for biopolymer films have been introduced to improve their bioactivities as well as properties. Gelatin/chitosan (G/C) blend films containing active guava leaf powder carbon dots (GL-CDs) at various levels (0–3%, *w*/*w*) were prepared by the solvent casting method and characterized. Thickness of the control increased from 0.033 to 0.041 mm when 3% GL-CDs were added (G/C-CD-3%). Young’s modulus of the resulting films increased (485.67–759.00 MPa), whereas the tensile strength (26.92–17.77 MPa) and elongation at break decreased (14.89–5.48%) as the GL-CDs’ level upsurged (*p* < 0.05). Water vapor barrier property and water contact angle of the film were enhanced when incorporated with GL-CDs (*p* < 0.05). GL-CDs had a negligible impact on film microstructure, while GL-CDs interacted with gelatin or chitosan, as determined by FTIR. The release of GL-CDs from blend films was more pronounced in water than in alcoholic solutions (10–95% ethanol). The addition of GL-CDs improved the UV light barrier properties and antioxidant activities of the resultant films in a dose-dependent manner. Thus, GL-CD-added gelatin/chitosan blend films with antioxidant activities could be employed as potential active packaging for the food industry.

## 1. Introduction

Plastic waste remains in the ecosystem and ultimately turns into micro- and nano-plastics, causing global plastic pollution. Three hundred MT of plastics are produced worldwide annually, and 1/3 have been reused or recycled. As a result, green or environmentally friendly packaging has become more crucial. Recently, there has been a rise in the usage of natural biopolymers, particularly chitosan and gelatin, in packaging to meet customer needs and solve ecological problems caused by the petroleum-based plastic counterpart [1,2].

Gelatin, a byproduct from animal and fish processing, is widely employed in foods and biodegradable packaging. It yields a flexible film with an efficient barrier property toward oxygen and aroma [3]. However, the presence of hydrophilic amino acids makes gelatin film have high water vapor transmission. Chitosan from shrimp shells has been used in packaging because of its low oxygen permeability, non-toxicity, biocompatibility and antimicrobial properties [4,5,6]. Its inferior water barrier and the flexibility of chitosan limit the application of food packaging. To overcome the drawbacks of single biopolymers, blend films have been made based on both chitosan and gelatin. The resulting blend film has better physiochemical and mechanical properties [7]. The composite films made using gelatin and chitosan had much improved properties than monomeric films [4,8]. To make active packaging, various approaches, e.g., the integration of nanoparticles, plant extract, fillers or additives, etc., have been implemented [9]. Nowadays, natural extracts have received greater attention for being incorporated into biopolymer-based films due to consumers’ preferences and safety concerns [10,11].

Active packaging effectively lengthens the shelf life of perishable food. Basically, the fillers or additives that can be absorbed or released from or into the surrounding environment or packaged food are incorporated into the package [12]. Plants are an excellent source of natural compounds possessing antioxidant activity [13]. Guava leaf has been traditionally utilized to cure a variety of ailments. The principal components of guava leaves are polyphenolics, e.g., flavonoids and tannins, which have antioxidant properties [14,15]. Ethanolic guava leaf extract possessed metal chelation, DPPH and ABTS radical-scavenging capabilities [16]. Plant extracts added to packaging not only act as antimicrobial and antioxidant agents but also influence the characteristics of the resulting packaging [9,17].

Carbon dots (CDs) have received a rising interest in food packaging since CDs have antioxidant or antimicrobial properties [3,18,19]. CDs are prepared from natural substances and provide their functions in active food packaging [20]. Inclusion of CDs into film-forming solutions could enhance their barrier properties and the antioxidant and antibacterial activities of food packaging [21,22,23,24]. Moreover, CDs made from natural materials are cost-effective, resourceful, environmentally friendly and biocompatible. Incorporation of Zn-CDs/Kohlrabi anthocyanin with carrageenan film [20] and green tea carbon dots with chitosan/gelatin blend films [3] resulted in superior barrier (mechanical and UV barrier) properties, antioxidant and antimicrobial activities compared to the control. Moreover, the addition of *Allium sativum* CDs to carrageenan/alginate films [25] led to slightly enhanced water vapor barrier properties. In addition, the inclusion of bioactive, eco-friendly fillers, especially CDs, effectively protects the foods from UV light. CDs also enhanced the functional properties of blend films, which can be used as a promising active packaging material [3,20,25,26]. Guava leaves can be obtained widely from tropical countries and can be used as a potential cheap source of CDs due to their abundance. Therefore, the use of GL-CDs as an active agent is an innovative approach for the preparation of active packaging to enhance the properties of blend films. However, no information on the packaging incorporated with GL-CDs exists. Thus, this study attempted to prepare and characterize films from gelatin/chitosan (G/C) blends incorporated with GL-CDs at different levels.

## 2. Results and Discussion

### 2.1. Properties and Characteristics of G/C Blend Film Incorporated without and with GL-CDs at Varying Levels

#### 2.1.1. Overall Appearance and Water Contact Angle

**Figure 1:**
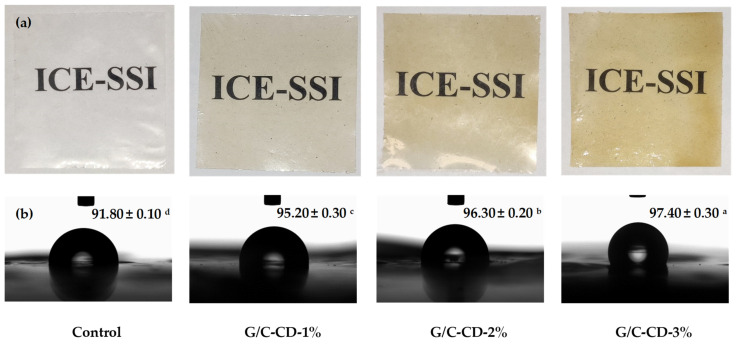
Photographs (**a**) and contact angle (**b**) of gelatin/chitosan blend films added without and with GL-CDs at different levels. Values are presented as mean ± SD (*n* = 3). Different lowercase superscripts in the same column indicate significant differences (*p* < 0.05). G/C: gelatin/chitosan blend films; GL-CDs: guava leaf carbon dots; Control: blend film without GL-CDs addition; 1%, 2% and 3% represent the percentage of GL-CDs added to blend films.

Photographs of G/C blend films containing GL-CDs at varying levels are illustrated in Figure 1. The control film was transparent and clear. GL-CD-added films were clear but had a slightly yellowish color. The yellow color became more pronounced with augmenting amounts of GL-CDs. Yellowish color might make the film undesirable in appearance. This could be the limitation of film incorporated with GL-CDs at high levels.

Different films showed varying hydrophobicities as indicated by different contact angles of water (θ_a(w)_) on the film surface. The film’s hydrophilic surface is indicated by θ_a(w)_ < 90°, while θ_a(w)_ > 90° denotes the hydrophobic surface [4]. The contact angle of blend films added without and with GL-CDs at varying amounts is illustrated in Figure 1. In general, a higher contact angle indicated the enhanced hydrophobicity. In Figure 1, the contact angle of all films augmented when the concentration of GL-CDs upsurged. The contact angle of the control film (91.80°) was less than that of GL-CD-added films (95.20–97.40°), suggesting greater hydrophilicity and less water resistance of the control film compared to CD-added films. Among all films, the G/C-CD-3% film showed a higher contact angle of 97.40°, reflecting enhanced hydrophobicity. GL-CDs, which are hydrophilic in nature, might undergo interaction via hydrogen bond with G/C blend film components. As a consequence, the polar groups became less available. Enhanced hydrophobicity was also documented for carrageenan/alginate films containing *Allium sativum* CDs [25]. Thus, the inclusion of GL-CDs influenced the hydrophobicity of G/C blend films.

#### 2.1.2. Thickness

Thickness is a key parameter determining the physical and mechanical properties of biopolymeric films [27]. G/C films had varying thicknesses, depending on the levels of GL-CDs, as presented in Table 1. Thickness was augmented when GL-CDs incorporated increased (*p* < 0.05). The G/C-CD-3% film possessed the highest thickness among all samples (*p* < 0.05). This was mostly due to the augmented content of solid matter present in GL-CDs. The upsurge of thickness in the blend films was plausibly caused by the inclusion of GL-CDs present in the matrix of the film, which reduced the compactness of the films as witnessed by the thicker films. The addition of green tea CDs in C/G blend films [3] and enoki mushroom CDs in gelatin/carrageenan films [28] resulted in a higher thickness than the control film. The result confirmed that CDs added to G/C films resulted in a disturbance of the ordered structure of the film matrix [9]. Thus, the inclusion and amounts of CDs directly influenced the thickness of G/C blend films.

#### 2.1.3. Mechanical Properties

Young’s modulus (YM), tensile strength (TS) and elongation at break (EAB) of G/C blend films incorporated without and with GL-CDs at varying levels are given in Table 1. Gelatin film generally has a more flexible structure with high elasticity, while chitosan has a semicrystalline structure, rendering it a stiff film [4,29]. For YM representing the stiffness or rigidity of the materials, the control film exhibited the lowest YM (485.67 MPa), but there was no difference in YM between the control and G/C-CD-1% films (*p* < 0.05). YM upsurged with an increasing amount of GL-CDs added to the film (*p* < 0.05). This indicated that CD-added films had increased stiffness and rigidity. The inclusion of CDs caused more resistance to tensile deformation of G/C blend films, plausibly due to enhanced inter-molecular interaction in the film network. Increased YM was also documented when Zn-CDs/Kohlrabi anthocyanin were added to carrageenan films [20] and when *Allium sativum* CDs were added to carrageenan/alginate films [25].

The control film had a higher TS (26.92 MPa) than the GL-CD-added films. TS of GL-CD-added films decreased gradually from 26.92 to 17.77 MPa as GL-CDs increased from 0 to 3% (*p* < 0.05). Lower TS was also found for tea CD-added chitosan films [30]. The decrease in TS of CD-added films coincided with the reduction in EAB value. The highest EAB was found in the control and G/C-CD-1% films (*p* < 0.05). The rest of the samples showed a decrease in EAB, and the lowest EAB was attained in the G/C-CD-3% film (*p <* 0.05). The decreases in TS and EAB were mostly due to the increased rigidity or stiffness of the film samples. The stiffer CD-added films possessed lower molecular orientation and less strain-hardening behavior upon tensile load deformation before fracture. This could result in a lower ultimate strength of the CD-added film compared to the control sample. The reduction in EAB could be because of the inclusion of foreign particles that might weaken the integrity of the film matrix [31]. Reduction in TS and EAB in the presence of GL-CDs, particularly at higher concentrations, could plausibly be related to the loss of the crystalline structure in the film [32].

However, some previous studies showed that CDs upsurged the TS of biodegradable films [3,26] since CDs had the ability to strengthen film networks. CDs from different sources might be varied, depending on compositions [25]. The introduction of bioactive natural fillers generally altered the mechanical characteristics of biopolymer-based films [20]. Structure and type of polymer, concentration, plasticizer and other additives majorly influence the mechanical strength and flexibility of the films [33]. Therefore, the incorporation of foreign components might impact the development of a proper film matrix.

Thermoplastic starch film added with graphene quantum dots [34] and carrageenan/alginate films added with *Allium sativum* CDs [25] exhibited similar results. Augmented aggregation of CDs at high concentrations in the film matrix might increase interaction between CDs, leading to lower interaction with polymer chains [25]. As a result, the mechanical properties of the blend films were affected by the inclusion of GL-CDs, and the amount of GL-CDs had a marked influence on the mechanical properties of G/C blend films.

#### 2.1.4. Water Vapor Permeability (WVP)

WVP is used to evaluate the rate at which water vapor passes through the film [4]. The films with low WVP are mostly preferred since moisture negatively induces changes in the quality of food products, especially the softening of texture as well as enhanced spoilage. The control film had the highest WVP value, while the G/C-CD-3% film exhibited the lowest WVP (*p* < 0.05) (Table 2). Thus, the addition of GL-CDs might effectively prevent the entry and penetration of water molecules. The inclusion of GL-CDs in the G/C blend film matrix plausibly provided a ‘tortuous pathway’ for molecules of water in the film network. Furthermore, interaction between gelatin and chitosan mediated by GL-CDs could reduce the hydrophilicity of the blend, as suggested by the increased water contact angle (Table 1). As a result, the film’s hydrophilicity decreased, and the reduction in WVP of the resulting films was achieved. However, CDs were documented to increase the WVP of biodegradable films added with CDs [3,26,28]. This might be due to differences in the CDs from different materials. Also, the formation of the film and polymers used might be different. Those factors affected the distribution and interaction of CDs in the film matrix, based on various polymers.

#### 2.1.5. Color

Lightness (L*-value) was highest for the control film, while the G/C-CD-3% film showed the lowest value (*p* < 0.05) (Table 2). This was related to the high transparency of the G/C blend film. GL-CDs were generally yellowish in color. The incorporation of GL-CDs into the G/C blend film slightly lowered the lightness and augmented the yellowness (b*-value). The control film exhibited lower a* (greenness) and b*-values than the film added with GL-CDs (*p* < 0.05). G/C-CD-3% film possessed a higher b*-value (yellowness), owing to the yellow color of GL-CDs. Similar results were found for CD-based biopolymer films [3,26,28]. A higher a* and ΔE*-values were also found for the G/C-CD-3% film (*p* < 0.05). The optical characteristics of films are frequently altered by the inclusion of active materials used to impart bioactivity [28]. The addition of natural extract modified the color of the resulting film to some extent [9]. Thus, the inclusion of GL-CDs into G/C blend films affected the color, especially yellowness, of the resultant films.

#### 2.1.6. Light Transmittance, Opaqueness and UV-Blocking Properties

At UV-200 nm, all films showed no light transmittance, and the reduced transmittance was also observed for GL-CDs containing film samples at 280 nm. Overall, the GL-CD-added films had low transmittance values in the UV region (200–400 nm) (Table 3), revealing a great barrier capacity against UV light. The UV barrier helps to maintain the quality of foods, especially high-fat foods prone to photocatalytic oxidation [28].

The inclusion of GL-CDs slightly changed the films to a yellowish color; however, the transparency of the film was not affected drastically (Figure 1). The opaqueness of the blend film upsurged as the concentration of GL-CDs was augmented (Table 3). The control film exhibited low opaqueness, whereas the G/C-CD-3% film showed the highest opaqueness. This reconfirmed that the G/C blend was tuned to be more opaque in the presence of GL-CDs. This change was in line with the increased b*-value.

**Figure 2:**
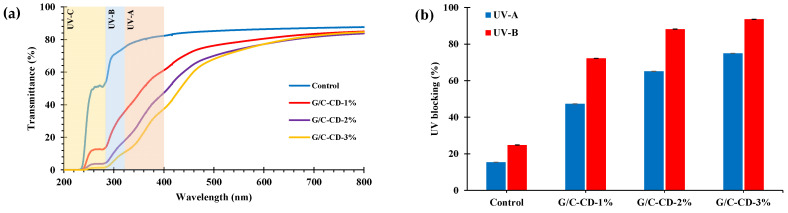
UV–visible light transmittance spectra (**a**) and UV-A (320−400 nm) and UV-B (280−320 nm) blocking properties (**b**) of gelatin/chitosan blend films added without and with GL-CDs at different levels. Bars represent the standard deviation (*n* = 3). Key: see Figure 1 caption.

Photolysis and photooxidation induced by UV light can deteriorate food quality. The UV-blocking ability of films is their main feature for preventing the loss of food quality mediated by UV light [35]. Additionally, UV light (below 400 nm) can accelerate the loss of organoleptic (odor, color and flavor) and nutritional quality of food [36]. The control film was more transparent than the others. It could block transmittance only for 15.39% of UV-A and 24.82% of UV-B light (Figure 2). Generally, the UV barrier properties of G/C film gradually augmented as the level of GL-CDs upsurged. For the GL-CD-added films, UV-A and UV-B were blocked by 47.31–75.03% and 72.28–93.61%, respectively. Thus, UV light was blocked by the addition of GL-CDs, particularly at high concentrations.

#### 2.1.7. ATR-FTIR Spectra

**Figure 3:**
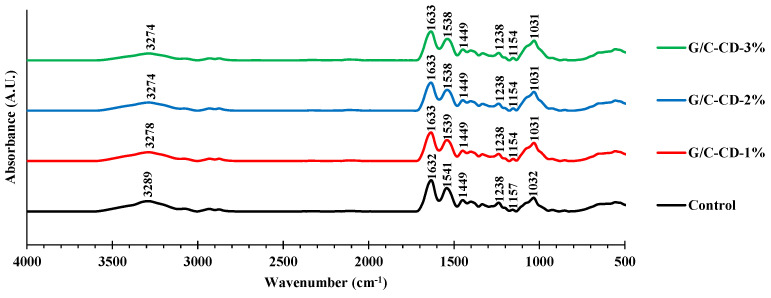
ATR-FTIR spectra of gelatin/chitosan blend films added without and with GL-CDs at different levels. Key: see Figure 1 caption.

The spectra of all G/C blend films are given in Figure 3. Amide-A bands at 3289, 3278, 3274 and 3274 cm^−1^ were found for the control film and those containing GL-CDs at 1, 2 and 3%, respectively. This band was related to O-H and N-H stretching vibrations [37]. Amide I (C=O stretch), Amide II (N–H bending) and Amide III (C-N stretching and N–H deformation from amide linkages) bands [4] appeared at various wavenumbers. Amide I was detected at 1632 cm^−1^ for the control and 1633 cm^−1^ for the GL-CD-added films. Amide II exhibited at 1541 cm^−1^ for the control, 1539 cm^−1^ for the G/C-CD-1% film and 1538 cm^−1^ for the G/C-CD-2% and G/C-CD-3% films. The peak observed at 1238 cm^−1^ for all films denotes Amide III [38]. The characteristic band at 1449 cm^−1^ for all films was ascribed to –OH group vibrations [39]. Wavenumbers of 1032 cm^−1^ for the control and 1031 cm^−1^ for GL-CD-added films were ascribed to the -OH group, primarily from plasticizer (glycerol) [40]. The peaks at 1157 and 1154 cm^−1^ for the control and GL-CD-added films were attributed to the C–OH stretch, a band for the saccharide of chitosan [39]. Similar peaks were documented for G/C blend films to those reported by Tagrida et al. [4]. The lower amplitude of all peaks in GL-CD-added films was noted compared to that of the control. However, some shifts of peaks, especially for Amide-A and Amide II, to lower wavenumbers were observed in GL-CD-added films compared to the control, indicating some interaction between carbon dots and G/C in the film matrix. Presumably, various active functional groups, including -OH, C=O and NH_2_, presented on the surface of CDs [3] could undergo H-bonding interactions with gelatin and chitosan molecules.

#### 2.1.8. Release Rate

**Figure 4:**
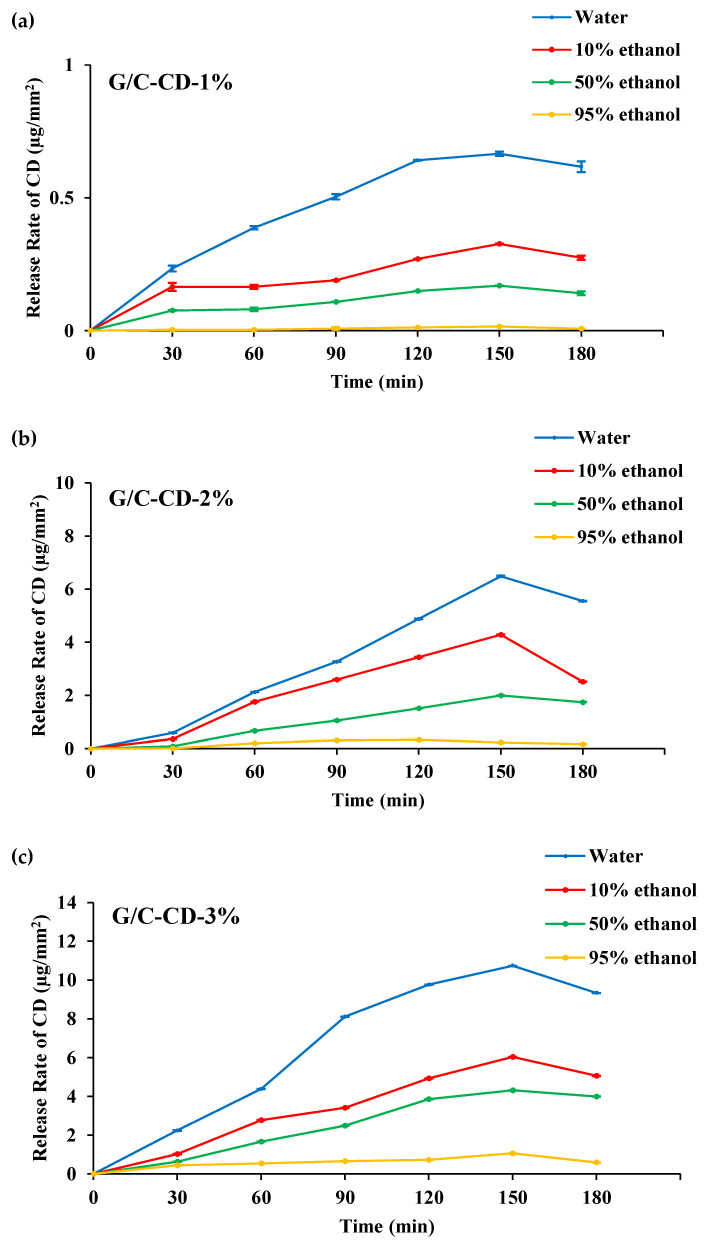
Release rate of GL-CDs from G/C-CD-1% (**a**), G/C-CD-2% (**b**) and G/C-CD-3% (**c**) films in different food simulants. Key: see Figure 1 caption.

The release of GL-CDs from G/C blend films into various simulant solutions is depicted in Figure 4. Among all the films, the release rate of GL-CDs from films in water and 10% ethanol was higher during the initial stage (30 min), and the release rate gradually increased after 30 min. However, compared to water and 10% ethanol, the release rate in 50 and 95% ethanol solutions was significantly lower, up to 180 min. When the concentration of ethanol upsurged, the release rate decreased. The release rate of GL-CDs from G/C blend films was concentration-dependent. The release rate of all films decreased after 150 min, when water and 10% ethanol were used as media. The release rate was governed by the solubility of the film matrix and the affinity of GL-CDs toward the tested solvents. Owing to the hydrophilic nature of G/C films, water readily passes through the film matrix and adequately hydrates the film, thus creating a looser structure and facilitating the removal of CDs from the film matrix [3]. Ethanol solutions, on the other hand, were unable to interact with gelatin/chitosan polymers effectively. This brought about the CDs remaining in the matrix, and their mobility and diffusion in closed systems were lowered. The enhanced release rate of GL-CDs in a hydrophilic environment could improve the bioactivity of the film when it comes into contact with the food surface.

#### 2.1.9. Antioxidant Activities

Antioxidant-releasing packaging can reduce the oxidation rate of foods and extend their shelf life [41]. The antioxidant properties of different G/C blend films are presented in Table 4. The control had lower DPPH and ABTS radical-scavenging activities (DPPH-RS-A and ABTS-RS-A, respectively) than GL-CD-added films (*p* < 0.05). RS-A of the control was plausibly due to the contribution of –NH_2_ or –OH groups of gelatin and amino groups of chitosan [42]. DPPH and ABTS-RS-As were upsurged as the amounts of GL-CDs rose (*p* < 0.05). The ability of GL-CDs to scavenge ABTS•+ and DPPH• was owing to the presence of oxygenated and amide functional groups on their surface [3]. Proton transfer from the -OH and amide groups of CDs was involved in the scavenging effect of GL-CDs [43]. CDs with a high surface area were able to provide the proton to radicals more effectively. In addition, most of the films exhibit lower antioxidant properties for DPPH-RS-A in comparison to ABTS-RS-A [3]. No ferric-reducing antioxidant power (FRA-P) and metal-chelating activity (MC-A) were detected for the control film. Tagrida et al. [4] stated that the G/C films did not have reducing power. Moreover, FRA-P and MC-A upsurged when the augmented level of GL-CDs was added to the blend films. The highest FRA-P and MC-A were found for G/C-CD-3% film, whereas the lowest FRA-P and MC-A were observed for G/C-CD-1% film. Thus, CDs with antioxidants that were extracted from the films could be released effectively from the film matrix and served as radical scavengers, as witnessed by both DPPH and ABTS-RS-As.

### 2.2. Characteristics of the Selected G/C Blend Film without and with 2% GL-CD

GL-CD-added film (G/C-CD-2%) with better mechanical and water barrier properties was chosen for further characteristics in comparison with the corresponding control counterpart (G/C-CD-0%).

#### 2.2.1. Microstructure, Elemental Mapping and Energy-Dispersive X-ray (EDX) Spectroscopy

**Figure 5:**
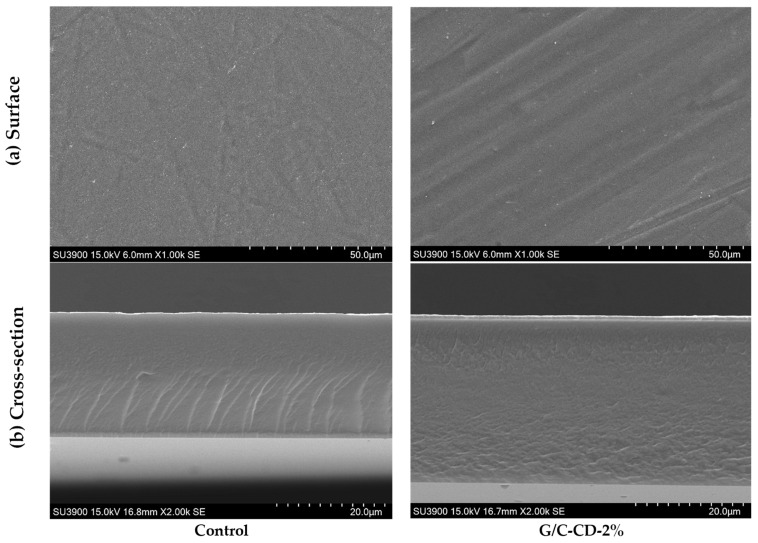
SEM surface (**a**) and cross-sectional images (**b**) of gelatin/chitosan blend film added without and with 2% GL-CDs. [Magnification: surface (1000×) and cross-section (2000×)]. Key: see Figure 1 caption.

Smooth, continuous and homogenous surface of both films without and with 2% GL-CDs addition was attained (Figure 5). Neither cracks nor bubbles were detected, suggesting that GL-CDs were compatible with the G/C film matrix. This indicated that a monolayer formed from the G/C blend could be formed via the casting method. This report was correlated with Khan et al. [3], who found that the incorporation of green tea CDs into the C/G blend film had no impact on the microstructure of the resulting film. However, the film became thicker in the presence of GL-CDs at 2%. The hydrophilic interaction of GL-CDs with biopolymers might induce entanglement in the way a protruded structure could be formed. SEM (cross-sections) images revealed no gaps, ruptures or holes, indicating that GL-CDs were uniformly dispersed in the G/C blend matrix. However, the presence of a slight fibrous structure in the control film disappeared when 2% GL-CDs were added. A similar phenomenon was also found [3,20].

**Figure 6:**
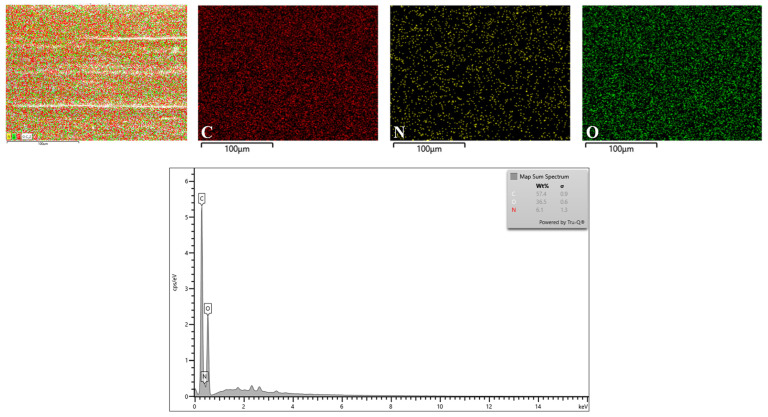
Elemental mapping and EDX spectra of G/C-CD-2% film. C, N and O represent carbon, nitrogen and oxygen, respectively. Key: see Figure 1 caption.

According to the EDX analysis (Figure 6), the primary components of the structural framework of the G/C-CD-2% film were carbon (C), nitrogen (N) and oxygen (O). Additionally, the mapping pictures showed that the dispersion of individual components was consistent across the surface. Thus, the GL-CDs were dispersed uniformly throughout the film matrix. The spectrum revealed that the weight and atomic ratios of C, O and N of the carbon dots were found to be 57.40:36.50:6.10, respectively. Among those elements, the percentage of C was higher than that of O and N. Thus, the availability of carbon in GL-CDs contributed to the additional content of carbon in the G/C-CD-2% film.

#### 2.2.2. Thermal Properties

**Figure 7:**
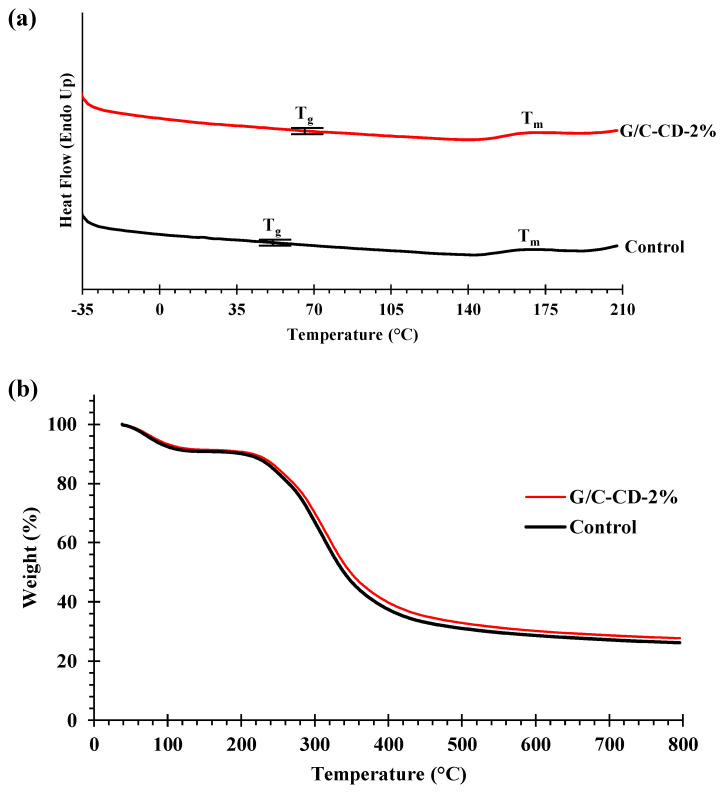
DSC thermograms (**a**) and thermogravimetric curves (**b**) of gelatin/chitosan blend films added without and with 2% GL-CDs. Key: see Figure 1 caption.

Differential scanning calorimetry (DSC) and thermogravimetric analysis (TGA) were implemented to evaluate the thermal-resistant properties of two blend film samples (control and G/C-CD-2%) as presented in Table 5, and the thermograms are shown in Figure 7a, b. T_g_ (glass transition temperature) of the control and G/C-CD-2% films were found at 52.47 and 64.97 °C, respectively. T_g_ is correlated with the segmental motion of polymer molecules in the amorphous phase [44]. The GL-CD-added film exhibited a higher T_g_ than the control. The inclusion of GL-CDs might enhance the interconnections of polymeric chains of the film to some degree, as evidenced by the FTIR result. This resulted in decreased chain mobility, thus augmenting the T_g_ and stiffness of the CD-added films (Table 1). The melting temperature (T_m_) appeared at 168.17 and 170.17 °C for the control and the G/C-CD-2% film, respectively. The disruption of the ordered or crystalline structures of films caused the melting transition [45]. Therefore, the thermal transition behavior of the G/C-CD-2% film was slightly different from that of the control.

Thermal degradation behavior was analyzed from 30 to 800 °C. The G/C blend film had weight loss at four different stages. The first stage (T_d1_) of weight loss (Δ*w*_1_ = 8.60–9.20%) for the control and G/C-CD-2% films took place at 54.99 and 51.54 °C, respectively. Weight loss at this stage was mostly attributed to the gradual loss of free water and other volatile constituents localized in the film matrix [46,47]. The second stage of weight loss (Δ*w*_2_ = 9.06–9.77%) was observed at T_d2_ of 223.33 and 221.49 °C for the control and G/C-CD-2% films, respectively. This was plausibly because of the loss of tightly bound water, glycerol, short-chain or low-MW molecules [4]. Δ*w*_3_ (51.34–52.24%) was observed at T_d3_ of 312.09 and 308.69 °C for the control and G/C-CD-2% films, respectively. Khan et al. [3] documented that this weight loss was related to the thermal decomposition of both chitosan and gelatin. The final stage of weight loss (Δ*w*_4_ = 2.61–3.31%) was found at T_d4_ of 622.14 and 613.29 for the control and G/C-CD-2% film, respectively. This weight loss might result from the decomposition of highly associated molecules with large MW in the matrix of the film. In all stages, the G/C-CD-2% film showed weight loss at a slightly lower temperature than the control film. The residual mass after heating was 26.18 and 27.69% for the control and G/C-CD-2% films, respectively. Thus, the GL-CDs’ incorporation had a slight impact on the thermal stability of the G/C blend film.

## 3. Materials and Methods

### 3.1. Chemicals

Chitosan (MW: ~2.1 × 10^3^ kDa; degree of deacetylation: ~82%; viscosity: 1000–2000 cps) was supplied by Marine Bioresources Co., Ltd. (Samut-Sakhon, Thailand). Fish skin gelatin (~250 bloom) was procured from Vihn Hoan (Dong Thap Province, Vietnam). Acetic acid was acquired from RCI lab scan Limited (Bangkok, Thailand). Other chemicals were supplied by Sigma-Aldrich, Inc. (St. Louis, MO, USA).

### 3.2. Preparation of Guava Leaf Powder (GLP) and Carbon Dots (GL-CDs)

Guava (*Psidium guajava* L.) leaves obtained from a plantation in Hat Yai, Thailand, were subjected to water cleaning and drying in a tray dryer (45 °C). Leaf powder was prepared with the aid of a high-speed grinder (Model 1000A, Qingdao, China) and finally sieved (80 mesh size). The obtained guava leaf powder was packed in airtight PE pouches, sealed/packed and kept at refrigerated temperatures until use.

Guava leaf carbon dots (GL-CDs) were prepared from guava leaf powder using a hydrothermal method [3]. Guava leaf powder (2%) was mixed with distilled water in a Teflon-lined cylinder and sealed in a stainless-steel reactor (20 mL). The suspension was heated at 200 °C (6 h) using a muffle furnace. After being cooled to 25 °C, the yellowish-brown mixture was filtered using a 100 μm Whatman filter paper with further centrifugation (5000× *g*, 15 min). Thereafter, the filtration of the supernatant was carried out with the aid of a filter (0.22 μm pore size, 13 mm diameter nylon filter, Membrane solutions, Jiangsu, China). Prior to analysis, the filtrate containing GL-CDs was kept at 4 °C.

The obtained GL-CDs solution was yellowish-brown in color but vividly turned blue under UV light. The sharp absorption peak (269.5 nm) and shoulder (364.5 nm) of GL-CDs were ascribed to the π–π* (C=C bond) or n–π* transitions (C=O bond), respectively. The diameters of the GL-CDs were in the range of 2.30–18.70 nm. The average diameter was 8.00 ± 1.90 nm. TEM images clearly displayed that most GL-CDs were spherical and monodisperse. The zeta potential of GL-CDs was -6.11 mV.

### 3.3. Study on Properties of G/C Blend Film Added with GL-CDs

Film-forming solution (FFS) made from gelatin and chitosan was prepared separately. Gelatin FFS was obtained by stirring gelatin (3.5%) in distilled water (60 °C) for 20 min. Chitosan FFS was attained by solubilizing chitosan (1%) in acetic acid (1%, *v*/*v*) [4]. Both FFSs at a 1:1 ratio (*v*/*v*) were mixed well and referred to as gelatin/chitosan FFS (G/C-FFS). Glycerol (30%, *w*/*w*) was employed as a plasticizer. To prepare the blend films loaded with GL-CDs, GL-CDs (0, 1, 2 and 3%, *w*/*w*) were added into the G/C-FFS with vigorous stirring (40 °C for 120 min). All FFSs were degassed with the aid of a sonicator (5 min). FFS (4 g) was cast evenly onto the rimmed silicone resin plate (5 × 5 cm^2^), followed by air drying (70% RH at 25 ± 0.5 °C) overnight and equilibrated in an environmental chamber (50 ± 5% RH at 25 ± 0.5 °C) for 48 h. Films were peeled and kept at 50% RH and 25 ± 0.5 °C for another 48 h. However, SEM, ATR-FTIR, DSC and TGA analyses were performed after film samples were conditioned in a desiccator containing silica gel (28–30 °C) for 7 days to obtain the most dehydrated films.

#### 3.3.1. Appearance and Water Contact Angle

Photos of all the films were taken by a smart phone camera (OnePlus Nord, Model AC2001, OnePlus Technology Co., Ltd., Shenzhen, China). The wettability of the selected films was examined, in which contact angle measurements were carried out by the sessile drop technique with the aid of a commercial contact angle meter (Dataphysics GmbH, Model OCA25, Filderstadt, Germany) [48].

#### 3.3.2. Thickness and Mechanical Properties

Thickness of all the films was determined using a micrometer (Mitutoyo Corp., Kawasaki-shi, Japan) at 10 randomly selected spots, and the average thickness was then computed. Measurements of Young’s modulus (YM), tensile strength (TS) and elongation at break (EAB) were carried out with the aid of the Universal Testing Machine (Lloyd Instruments, Hampshire, UK) [45]. Ten film samples (20 × 40 mm^2^) with an initial grip length of 30 mm were clamped, and testing was carried out using a load cell of 200 N and a cross-head speed of 30 mm/min.

#### 3.3.3. Water Vapor Permeability

Water vapor permeability (WVP) of the films was evaluated [4]. Film samples were placed and sealed over the aluminum permeation cup containing dried silica and sealed tightly. After keeping in an environmental chamber (25 ± 0.5 °C; 50% RH), the cup was weighed, and the WVP was computed every 1 h up to 10 h.

$$WVP\;\left(g\cdot m\cdot m^{-2}\cdot s^{-1}\cdot {Pa}^{-1}\right)=wlA^{-1}t^{-1}(P_{2}-P_{1}{)}^{-1}$$

where *w* stands for the weight gain of the cup (g); *l* denotes the film thickness (m); *A* denotes the exposed film area (m^2^); *t* is the time of gain (s); (*P*_2_* − P*_1_) stands for vapor pressure difference across the film (Pa).

#### 3.3.4. Color, Light Transmittance, Opaqueness and UV-Blocking Properties

Color of the films were measured using a colorimeter (Hunterlab, Reston, VA, USA) [4]. ΔE* (total color difference) was computed.

$$\Delta E^{*}=\sqrt{(\Delta L^{*}{)}^{2}+(\Delta a^{*}{)}^{2}+(\Delta b^{*}{)}^{2}}$$

where ΔL*, Δa* and Δb* are the differences in color parameters of the film in comparison to those of the white standard (L* = 91.55, a* = −1.81 and b* = −1.21).

Light transmission of films was evaluated in the range of 200–800 nm with a UV–visible spectrophotometer (Model UV-1800, Shimadzu, Kyoto, Japan) [45]. The opaqueness value was computed.

$$Opaqueness\;value=\frac{{-\log\;T}_{600}}{X}$$

where *T*_600_ represents fractional transmittance at 600 nm and *X* represents film thickness (mm).

The UV barrier property toward *UV-A* (320–400 nm) and *UV-B* (280–320 nm) was determined following the procedure of Koutchma et al. [49], in which the percentage blocking of *UV-A* and *UV-B* was computed using the following equations:

$$UV\text{-}A\;blocking\left(\%\right)=100-\frac{\int_{320}^{400}T\left(\lambda \right)d\lambda }{\int_{320}^{400}d\lambda }\;UV\text{-}B\;blocking\left(\%\right)=100-\frac{\int_{280}^{320}T\left(\lambda \right)d\lambda }{\int_{280}^{320}d\lambda }$$

where *T*(*λ*) is the average transmittance of film at the tested wavelength *λ* and *dλ* is the bandwidth of film.

#### 3.3.5. ATR-FTIR Spectra

FTIR spectra of all the films were analyzed with a Bruker FTIR spectrometer (Model Equinox 55, Bruker Co., Ettlingen, Germany). It was equipped with an ATR platinum crystal at 25 °C, as detailed by Tagrida et al. [4].

#### 3.3.6. Release Test

The amount of GL-CDs released from the G/C blend films through food stimulants was monitored [3]. Films (2.5 × 2.5 cm^2^) were immersed into 20 mL of various food simulant solutions (water, 10%, 50% and 95% ethanol for mimicking aqueous and lipid-based foods). The testing samples were constantly shaken using a shaker (Heidolph UNIMAX 1010, Schwabach, Germany) for 180 min (25 °C). Furthermore, the solution (1 mL) was taken, and the absorbance at 269.5 nm was read using a UV–visible spectrophotometer (Model UV-1800, Shimadzu, Kyoto, Japan) at 0, 30, 60, 90, 120, 150 and 180 min.

#### 3.3.7. Antioxidant Activities

Small pieces of films (100 mg) were mixed with 80% methanol (10 mL) and stirred for 18 h [4]. The mixture was centrifuged (8000× *g*, 20 min). The obtained supernatants were examined for DPPH and ABTS radical-scavenging activities (DPPH-RS-A and ABTS-RS-A, respectively), ferric-reducing antioxidant power (FRA-P) and metal chelating activity (MC-A).

### 3.4. Characterization of the Selected G/C Blend Film without and with 2% GL-CDs

Control film and the film added with 2% GL-CDs having satisfactory properties were further analyzed.

#### 3.4.1. Microstructure and Elemental Mapping

Microstructures of different G/C blend films were examined using a scanning electron microscope (SEM) (Hitachi, SU3900, Tokyo, Japan) at 15 kV as tailored by Martucci and Ruseckaite [50]. By using double-sided adhesive tape, cryo-fractured films with a gold coating were visualized at a magnification of 1000× for the surface and 2000× for the cross-section.

Elemental mapping and the EDX spectrum were examined with the aid of SEM and an electron-dispersive X-ray spectroscope (EDX) [51]. Elemental analysis was performed on the surface of the film (G/C-CD-2%).

#### 3.4.2. Differential Scanning Calorimetry (DSC) and Thermogravimetric Analysis (TGA)

DSC spectra of films were examined using a DSC-3+ (Mettler Toledo, Greifensee, Switzerland). The film (5 mg) was placed in an aluminum pan and sealed, and the scanning from −30 to 210 °C was conducted at 10 °C/min [4]. TGA was analyzed by scanning the film samples at 10 °C/min for 30 to 800 °C (TGA-8000, Perkin Elmer, Norwalk, CT, USA), and nitrogen was used as the purge gas (20 mL/min) [45]. The empty pan was used as a reference.

### 3.5. Statistical Analysis

A completely randomized design was adopted for this entire study. A one-way ANOVA was performed, and a Duncan’s multiple range test was used to compare means. Data analysis was performed using the SPSS package (SPSS 27.0 for Windows, SPSS Inc., Chicago, IL, USA).

## 4. Conclusions

Gelatin/chitosan blend films incorporated with active guava leaf powder carbon dots (GL-CDs) were developed by the solvent casting method, and their properties were governed by the levels of GL-CDs added. The incorporation of GL-CDs enhanced the water vapor barrier, UV-barrier and antioxidant properties of the blend films. Thus, the enhanced barrier properties of the active GL-CD-based gelatin/chitosan blend film could be achieved. Overall, the G/C-CD-2% film showed relatively better properties and characteristics than its control counterpart. GL-CDs at 2% were therefore suggested to be added to the film to yield active packaging for food applications, particularly for lowering lipid oxidation in packaged foods.

## Figures and Tables

**Table 1:** Thickness, Young’s modulus (YM), tensile strength (TS) and elongation at break (EAB) of gelatin/chitosan blend films added without and with GL-CDs at different levels.

Film Samples	Thickness (mm)	YM (MPa)	TS (MPa)	EAB (%)
Control	0.033 ± 0.001 ^d^	485.67 ± 14.22 ^c^	26.92 ± 0.73 ^a^	14.89 ± 0.09 ^a^
G/C-CD-1%	0.037 ± 0.002 ^c^	542.00 ± 61.61 ^bc^	22.16 ± 1.41 ^b^	14.06 ± 1.36 ^a^
G/C-CD-2%	0.039 ± 0.001 ^b^	586.00 ± 9.54 ^b^	20.25 ± 0.39 ^c^	12.08 ± 1.30 ^b^
G/C-CD-3%	0.041 ± 0.002 ^a^	759.00 ± 2.00 ^a^	17.77 ± 0.14 ^d^	5.48 ± 0.73 ^c^

Values are presented as mean ± SD (*n* = 3). Different lowercase superscripts in the same column indicate significant differences (*p* < 0.05). G/C: gelatin/chitosan blend films; GL-CDs: guava leaf carbon dots; Control: blend film without GL-CDs addition; 1%, 2% and 3% represent the percentage of GL-CDs added to blend films.

**Table 2:** Water vapor permeability and color of gelatin/chitosan blend films added without and with GL-CDs at different levels.

Film Samples	WVP ** (×10^−11^ g·m·m^−2^·s^−1^·Pa^−1^)	L*	a*	b*	ΔE*
Control	1.01 ± 0.03 ^a^	89.34 ± 0.12 ^a^	−1.95 ± 0.04 ^b^	−0.34 ± 0.07 ^d^	2.38 ± 0.12 ^d^
G/C-CD-1%	0.72 ± 0.11 ^b^	82.27 ± 1.64 ^b^	−1.87 ± 0.24 ^b^	10.11 ± 0.97 ^c^	14.65 ± 1.79 ^c^
G/C-CD-2%	0.67 ± 0.03 ^b^	79.18 ± 0.90 ^c^	−1.46 ± 0.11 ^a^	17.33 ± 0.87 ^b^	22.3 ± 1.21 ^b^
G/C-CD-3%	0.65 ± 0.09 ^b^	76.11 ± 0.80 ^d^	−1.39 ± 0.29 ^a^	26.48 ± 1.82 ^a^	31.7 ± 1.98 ^a^

Values are presented as mean ± SD (*n* = 3). Different lowercase superscripts in the same column indicate significant differences (*p* < 0.05). ** WVP was examined at 50% RH and 25 °C. L*, a*, b* and ΔE* represent lightness, redness/greenness, yellowness/blueness and color difference, respectively; G/C: gelatin/chitosan blend films; GL-CDs: guava leaf carbon dots; Control: blend film without GL-CDs addition; 1%, 2% and 3% represent the percentage of GL-CDs added to blend films.

**Table 3:** Light transmittance and opaqueness value of gelatin/chitosan blend films added without and with GL-CDs at different levels.

Film Samples	Light Transmittance (%) at Different Wavelengths (nm)								Opaqueness Value
	200	280	350	400	500	600	700	800	
Control	0.04 ± 0.01 ^a^	50.34 ± 0.79 ^a^	77.46 ± 2.07 ^a^	80.50 ± 1.85 ^a^	83.91 ± 1.51 ^a^	85.3 ± 1.27 ^a^	86.25 ± 1.08 ^a^	86.95 ± 0.90 ^a^	2.11 ± 0.16 ^b^
G/C-CD-1%	0.02 ± 0.02 ^ab^	12.88 ± 0.54 ^b^	46.98 ± 1.72 ^b^	61.78 ± 2.20 ^b^	77.08 ± 2.22 ^b^	81.42 ± 2.05 ^b^	84.38 ± 1.75 ^a^	85.84 ± 1.55 ^a^	2.45 ± 0.28 ^b^
G/C-CD-2%	0.01 ± 0.01 ^b^	4.10 ± 0.07 ^c^	28.87 ± 0.03 ^c^	47.42 ± 0.06 ^c^	69.58 ± 0.30 ^c^	76.77 ± 0.30 ^c^	81.26 ± 0.30 ^b^	83.48 ± 0.29 ^b^	2.99 ± 0.05 ^a^
G/C-CD-3%	0.01 ± 0.01 ^b^	0.82 ± 0.44 ^d^	14.41 ± 2.70 ^d^	32.01 ± 3.29 ^d^	63.65 ± 1.80 ^d^	73.84 ± 0.92 ^d^	79.70 ± 0.40 ^b^	82.40 ± 0.31 ^b^	3.20 ± 0.19 ^a^

Values are presented as mean ± SD (*n* = 3). Different lowercase superscripts in the same column indicate significant differences (*p* < 0.05). G/C: gelatin/chitosan blend films; GL-CDs: guava leaf carbon dots; Control: blend film without GL-CDs addition; 1%, 2% and 3% represent the percentage of GL-CDs added to blend films.

**Table 4:** Antioxidant activities of gelatin/chitosan blend films added without and with GL-CDs at different levels.

Film Samples	DPPH-RS-A (µmol TE/100 g Sample)	ABTS-RS-A (µmol TE/100 g Sample)	FRA-P (µmol TE/100 g Sample)	MC-A (µmol EE/100 g Sample)
Control	14.42 ± 1.54 ^d^	62.00 ± 0.50 ^d^	ND	ND
G/C-CD-1%	87.67 ± 2.14 ^c^	196.50 ± 14.73 ^c^	60.41 ± 8.86 ^c^	29.08 ± 0.34 ^c^
G/C-CD-2%	95.95 ± 0.93 ^b^	358.50 ± 25.86 ^b^	186.97 ± 11.59 ^b^	33.96 ± 2.52 ^b^
G/C-CD-3%	99.87 ± 0.46 ^a^	567.50 ± 2.00 ^a^	312.03 ± 2.96 ^a^	51.34 ± 2.69 ^a^

Values are presented as mean ± SD (*n* = 3). Different lowercase superscripts in the same column indicate significant differences (*p* < 0.05). DPPH-RS-A: DPPH radical-scavenging activity; ABTS-RS-A: ABTS radical-scavenging activity; FRA-P: ferric reducing antioxidant power; MC-A: metal chelating activity. G/C: gelatin/chitosan blend films. GL-CDs: guava leaf carbon dots. Control: blend film without GL-CDs addition; 1%, 2% and 3% represent the percentage of GL-CDs added to blend films.

**Table 5:** Glass transition (T_g_, °C), melting (T_m_, °C), thermal degradation temperatures (T_d_, °C), weight loss (Δ*w*, %) and residue (%) of gelatin/chitosan blend films added without and with 2% GL-CDs.

Film Samples	T_g_	T_m_	Δ_1_		Δ_2_		Δ_3_		Δ_4_		Residue
			T_d1_, Onset	Δ*w*_1_	T_d2_, Onset	Δ*w*_2_	T_d3_, Onset	Δ*w*_3_	T_d4_, Onset	Δ*w*_4_	
Control	52.47	168.17	54.99	9.20	223.33	9.77	312.09	52.24	622.14	2.61	26.18
G/C-CD-2%	64.97	170.17	51.54	8.60	221.49	9.06	308.69	51.34	613.29	3.31	27.69

Δ_1_, Δ_2_, Δ_3_ and Δ_4_ denote the first, second, third and fourth stage of weight loss, respectively, of films during the TGA heating scan (30–800 °C). G/C: gelatin/chitosan blend films; GL-CDs: guava leaf carbon dots; Control and G/C-CD-2% represent the films without and with the addition of 2% GL-CDs, respectively.

## Data Availability

The data used to support the findings of this study can be made available by the corresponding author upon request.
